# Future Scenarios for Academic Medicine Should Include Other Health Disciplines

**DOI:** 10.1371/journal.pmed.0020282

**Published:** 2005-08-30

**Authors:** Patrick McGrath

**Affiliations:** **1**Dalhousie UniversityHalifax, Nova ScotiaCanada

With reference to Awasthi and colleagues' Policy Forum [1], International Campaign to Revitalise Academic Medicine (ICRAM) has done an excellent service by provoking thought about the future of academic medicine by outlining five scenarios. However, all of the scenarios are seriously deficient because they do not sufficiently incorporate in their vision other health disciplines and professions. Increasingly, translational research and knowledge transfer depend on interdisciplinary teams. Good patient care requires team approaches. Academic medicine is realizing the value of collaboration and is opening itself to disciplines such as nursing, psychology, occupational therapy, and physiotherapy. In addition, disciplines such as health informatics are vital. Envisioning the successful future of academic medicine requires collegial inclusion of other health-related disciplines.
